# Astragaloside IV Derivative (LS-102) Alleviated Myocardial Ischemia Reperfusion Injury by Inhibiting Drp1^Ser616^ Phosphorylation-Mediated Mitochondrial Fission

**DOI:** 10.3389/fphar.2020.01083

**Published:** 2020-09-17

**Authors:** Li Chen, Xiao-Yi Chen, Qian-Long Wang, Si-Jin Yang, Hua Zhou, Li-Sheng Ding, Lin-Sen Qing, Pei Luo

**Affiliations:** ^1^State Key Laboratories for Quality Research in Chinese Medicines, Macau University of Science and Technology, Macau, China; ^2^Department of Cardiac Encephalopathy, Traditional Chinese Medicine Hospital Affiliated to Southwest Medical University, Luzhou, China; ^3^Chengdu Institute of Biology, Chinese Academy of Sciences, Chengdu, China

**Keywords:** astragalosidic acid, myocardial ischemia reperfusion, mitochondrial fission, GSK-3β, Drp1 phosphorylation

## Abstract

Our previous studies showed that Astragaloside IV derivative (LS-102) exhibited potent protective function against ischemia reperfusion (I/R) injury, but little is known about the mechanisms. Mitochondrial fission regulated by dynamin-related protein1 (Drp1) is a newly recognized determinant of mitochondrial function. This study aimed to investigate the protection of LS-102 on mitochondrial structure and function by regulating the activity of Drp1 using models of H9c2 cardiomyocyte injury induced by hypoxia-reperfusion (H/R), and rat heart injury induced by I/R. The results showed that LS-102 significantly decreased apoptosis, levels of ROS, CK, LDH, and calcium, upregulating MMP, and the Bax/Bcl-2 ratio in cardiomyocytes during I/R injury. Furthermore, LS-102 prevented I/R-induced mitochondrial fission by decreasing Drp1’s mitochondrial localization through decreasing the phosphorylation of Drp1 at Ser616 (Drp1^Ser616^) and increasing the phosphorylation of Drp1 at Ser637 (Drp1^Ser637^) in H9c2 cells. Importantly, we also robustly confirmed Drp1^Ser616^ as a novel GSK-3β phosphorylation site. GSK-3β-mediated phosphorylation at Drp1^Ser616^ may be associated with mitochondrial fission during I/R of cardiomyocytes. In conclusion, LS-102 exerts cardio protection against I/R-induced injury by inhibiting mitochondrial fission via blocking GSK-3β-mediated phosphorylation at Ser616 of Drp1.

## Introduction

Ischemic heart disease (IHD) is one of the biggest killers in the world (Nowbar et al., 2019). Early reperfusion therapy is now standard treatment for patients with acute myocardial infarction. However, myocardial ischemia reperfusion (I/R) further aggravates cell apoptosis, inflammation, and the production of reactive oxygen species (ROS) and calcium, which can lead to heart conditions (Dominguez-Rodriguez et al., 2014). How to alleviate I/R injury is a key subject for the treatment of IHD (Jennings, 2013).

The study investigated mitochondrial dysfunction is a key factor of pathogenesis, examining whether the mitochondrial function is based on the stabilization of the mitochondrial membrane structure (Turer and Hill, 2010). Recent studies have highlighted that an imbalance of mitochondrial dynamics plays a role in the pathological process of disease (Kuzmicic et al., 2011). During I/R, massive fission of mitochondria causes damaged to the function and structure of mitochondria (Sharp et al., 2014). Concomitantly, the mPTP of myocardial mitochondria is opened and then cytochrome c release. Dynamin-related protein1 (Drp1) is the predominant molecular mediator of mitochondrial fission, which is recruited to fission sites on the mitochondrial outer membrane and initiates mitochondrial fission. Drp1-mediated mitochondrial fission not only aggravates mitochondrial dysfunction but also leads to cell death (Hu et al., 2017). Inhibiting the activity of Drp1 is a therapeutic target to reduce I/R-induced injury (Tao et al., 2018).

Dynamin-related protein1 activity is modulated by posttranslational modifications including phosphorylation, ubiquitination, sumoylation, S-nitrosylation, and so on. Phosphorylation of Drp1 plays a crucial role in Drp1 activity regulation (Jahani-Asl and Slack, 2007). Most studies show that ser616 phosphorylation promotes the translocation of Drp1 to the mitochondrial outer membrane, However, Ser637 phosphorylation reverses this process (Taguchi et al., 2007). Glycogen synthase kinase-3β (GSK-3β), a serine/threonine protein kinase, plays a critical role in mitochondrial bioenergetics, mitochondrial permeability, mitochondrial motility, and mitochondrial apoptosis. Recently, mounting evidence shows that GSK-3β can control cell fate by regulating mitochondrial morphology (Gomez et al., 2008). Previous studies have indicated that GSK-3β interacted with Drp1 in yeast and mammals (Hong et al., 1998; Chen et al., 2000). The activated GSK-3β increases Drp1 GTPase activity to cause mitochondrial fragmentation in the pathogenesis of Alzheimer’s disease (AD; Yan et al., 2015). Furthermore, it is unclear whether GSK-3β can contribute to mitochondrial fragmentation in the pathogenesis of I/R through Drp1-dependent mechanisms.

**FIGURE 1:**
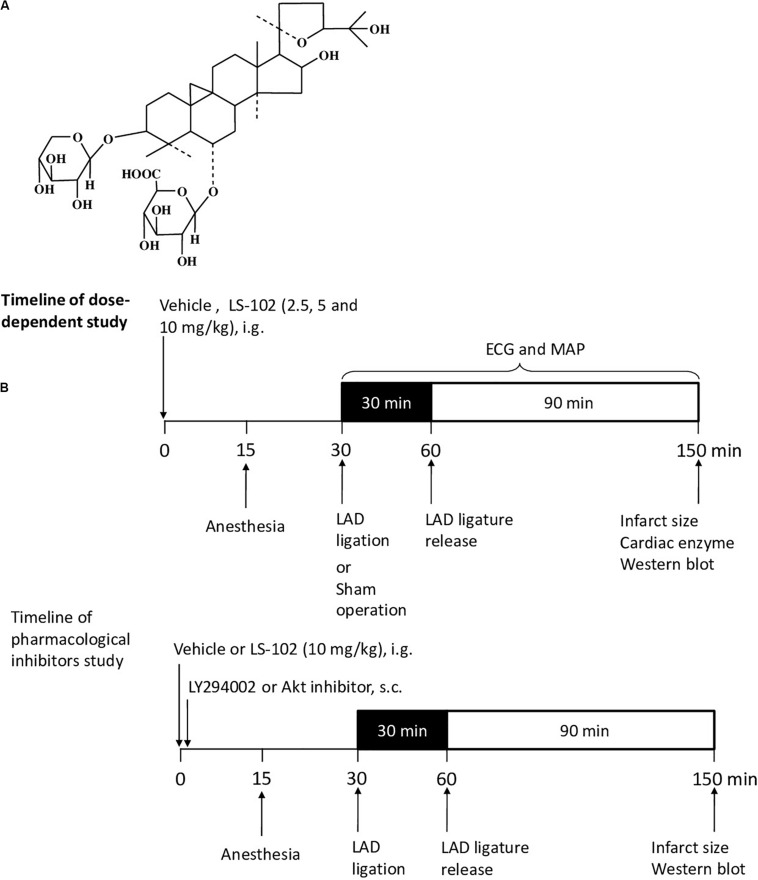
Outline of the experimental protocol to assess the cardioprotective effect of LS-102 on the model of myocardial I/R-induced rat injury. **(A)** Molecular structure of LS-102. **(B)** Protocols of dose-dependent study and pharmacological inhibitors study. Rats were pretreated with vehicle or LS-102, followed by 30 min of ischemia and then 90 min of reperfusion. Rat hearts were collected for TTC and H&E staining, the rat serum samples were also collected to detect the activities of CK and LDH.

Astragali Radix, which is commonly used in traditional Chinses medicine, has been included in the Chinese Pharmacopeia (Chinese Pharmacopoeia Commission, 2015). Studies have shown that Astragali Radix has pharmacological effects on cardiovascular diseases and immune regulation (Sun et al., 2019). Astragaloside IV (AS-IV), the major pharmacologically active ingredient of Astragali Radix, protects the internal organs from physical damage by exerting antioxidant, anti-inflammatory, and anti-apoptotic properties (Ren et al., 2013). Previous studies have demonstrated that AS-IV has a potent protective effect on I/R injury by activating the PI3K/Akt/GSK-3β pathway (Zhao et al., 2012). Astragalosidic acid (LS-102) is a new water-soluble derivative of astragaloside IV that has been synthesized by our team (Qing et al., 2019). However, the principle of the cardioprotective properties of LS-102 through mitochondrial dynamics in I/R injury has not been investigated. Figure 1A shows the structure of this new compound. In this study, two well-established models of I/R on SD rats and H9c2 cells were used to investigate the effect of LS-102 on mitochondrial dysfunction and the dynamics of cardiomyocytes.

## Materials and Methods

### Animals and Experimental Design

Male Sprague-Dawley rats (180–220 g) were purchased from the Laboratory Animal Services Center, of the Chinese University of Hong Kong (Hong Kong). The animals were cultured with a standard diet in accordance with the Institutional Guidelines and Animal Ordinance, in the stable environment of an animal room of a specific pathogen-free (SPF) laboratory (12 h light/dark cycle, 40–50% humidity at 20–23°C). Sixty rats were randomized into six groups (*n* = 10), respectively as follows: Sham, I/R, Verapamil, Low LS-102, Mid LS-102, and High LS-102. Distilled water at 8 ml/kg was administered intragastrically as a vehicle in Sham and I/R groups. LS-102 was dissolved in distilled water and administered intragastrically at 2.5, 5 and 10 mg/kg 30 min before LAD ligation. Verapamil as a positive control was given intravenously at 0.2 mg/kg 30 min before LAD ligation (Figure 1B). LY294002 (0.25 mg/kg, s.c. Sigma, St. Louis, MO, United States) or Akt inhibitor IV (4 mg/kg, s.c. Sigma, St. Louis, MO, United States) was given immediately after the oral administration of LS-102 (10 mg/kg). Thus, fifty rats were randomized into five groups (*n* = 10), respectively as follows: Sham, I/R, LS-102, LY294002+ LS-102, and Akt inhibitor IV+ LS-102. Out of 10 rats in each group, 6 were used for ventricular function and infarct size measurement and 4 rats for western blot analysis (Figure 1B).

### Myocardial I/R Injury Rat Model

The rat tests were performed according to the previous description with our modifications (Buerke et al., 1995). In brief, rats were anesthetized with pentobarbital sodium (i.p., 70 mg/kg body weight) and underwent endotracheal intubation and mechanical ventilation. After thoracotomy, rats were operated on, using an open-chest left anterior descending (LAD) coronary artery ligation, 30 min after ischemia, and then 90 min after reperfusion. The sham group rats were subjected to all the procedures except that the LAD ligation was not tightened. The vehicle or drugs were administered 30 min before ischemia via single oral administration. The right carotid artery was isolated and a Millar catheter (Millar Instruments, Inc., Houston, TX, United States) was inserted into the right carotid artery. Using a Power Lab (AD Instruments Pty Ltd., Castle Hill, Australia), mean arterial pressure (MAP) was recorded from the Millar catheter. An electrocardiogram (ECG) in lead II was recorded through needle electrodes attached to the limbs. The heart rate and ST-segment elevation were calculated off-line.

### TTC and H&E Staining

Hearts were washed with ice-cold PBS after excising and then weighed. The infarct size of the whole left ventricle was determined using 2,3,5-triphenyltetrazolium as described previously (Zhou et al., 2011). The left ventricle was sliced into transverse sections of five 2–3-mm-thick and then stained in 1% TTC solution in PBS. Images of the stained sections were captured by leica DFC480. The Infarct area of each slice was measured using planimetry method and Image J software. The histological changes in the heart tissue of the ischemic penumbra part in different groups were observed by H&E staining. Each heart was sectioned into a transverse (coronal) of four equal sections and then fixed directly in 10% buffered formalin in a cassette. Sections were dehydrated in increasing concentrations of ethanol, then cleared with xylene and embedded in paraffin, and then prepared for 3 μm sections.

### Cell Culture and *in vitro* Cardiomyocyte Model of H/R

The H9c2 cells (CRL1446, ATCC, United States) were cultured in Dulbecco’s Modified Eagle Medium (DMEM), supplemented with 10% fetal bovine serum (FBS) and 1% v/v penicillin/streptomycin (Gibco, Oklahoma, United States). Then the cells were seeded in tissue-culture dishes at 37°C with 5% humidified atmosphere CO_2_. LS-102 synthesized by our team was dissolved in Dimethyl sulfoxide (DMSO) and stored as 50 μM store solution at −20°C. H9c2 cells were cultured in saturated glucose-free DMEM and exposed to Whitley H35 Hypoxystation (0.1% O_2_, 5% CO_2_, and 94.9% N_2_) at 37°C for 12 h (hypoxia period) to induce hypoxia/reoxygenation (H/R) injury. Subsequently, the cells were cultured in DMEM supplemented with 10% FBS under normoxia (21% O_2_) for 2 h (reoxygenation period).

### Cell Viability and Mitochondrial Viability

The cell viability was detected by the method of 3-(4,5-dimethylthiazol-2-yl)-2,5-diphenyltetrazolium bromide (MTT, Molecular Probes, United States). A Multi-Mode Detection Platform (SpectraMax Paradigm, Molecular Devices, United States) was used to detect the absorbance at 570/650 nm. The mitochondrial viability assay kit (Abcam, United States) was used to observe the mitochondrial viability according to the manufacturer’s recommendations. The fluorescence intensity was detected at 590 or 550 nm to the direction of excitation by using the Multi-Mode Detection Platform.

### Lactate Dehydrogenase Activity, Intracellular Creatine Kinase Activity, Superoxide Dismutase (SOD) Activity, and ATP Content Assay

After treatment, the supernatant medium was collected to measure lactate dehydrogenase (LDH) activity using commercial assay kits (Beyotime Institute of Biotechnology, Beijing, China) according to the manufacturer’s recommendations. An absorbance of 450/660 nm was calculated by a Multi-Mode Detection Platform. According to the manufacturers’ respective protocols, the treated cells were collected using the creatine kinase (CK) test kit (Biovision, CA, United States) and the superoxide dismutase (SOD) test kit (Beijing Beiyang Institute of Biotechnology, China) to detect CK and SOD activities. An absorbance of 450 nm was evaluated by a Multi-Mode Detection Platform. 1 × 10^4^ H9c2 cells/well were seeded in the dark 96-well plate. After treatment, a luminescent ATP detection assay kit (Abcam, Cambridge, United Kingdom) was employed to detect the ATP level according to the manual.

### Apoptosis Analysis With a Flow Cytometer

Cell apoptosis was detected using FITC Annexin V Apoptosis Detection Kit (BD, Franklin Lakes, NJ, United States) according to the manufacturer’s recommendations. Briefly, H9c2 cells were cultured in a six-well plate. After H/R treatment, cells were collected and washed three times with PBS and then suspended in 1× binding buffer containing FITC-labeled Annexin V and propidium iodide (BD, Franklin Lakes, NJ, United States) in dark at room temperature. The fluorescence intensity was examined by BD FACSAria III (BD Biosciences, Franklin Lakes, NJ, United States). The intensity of each group (1 × 10^4^ cells) was calculated as representation. Apoptosis of each group was analyzed by Flow J software.

### Evaluation of Oxygen Cellular Oxygen Consumption Rate

Seahorse XF8 device (Seahorse Bioscience, United States) was used to measure the oxygen consumption rate (OCR). Briefly, cells (5000 cells/well) were cultured in the XFp cell culture plates. After experiencing H/R, cells were incubated in the 37°C non-CO_2_ incubator with 180 μl assay medium (XF Base Medium, 5.5 mM glucose, 1 mM pyruvate, and 2 mM L-glutamine, pH 7.4) according to the manufacturers’ recommendations. The sensor cartridge of the XFp analyzer was calibrated for 24 h in the non-CO_2_ incubator at 37°C. During the sensor calibration, the OCR of cells was normalized for total protein/well.

### ROS, Calcium and Mitochondrial Membrane Potential (MMP) Determination

The CM-H2DCFDA dye, Fluo-4 AM calcium dye (Molecular Probes, United States), and JC-1 dye (Molecular Probes, United States) were employed to assess ROS, calcium, and MMP, respectively. The fluorescent images of calcium and JC-1 were captured by a confocal microscope. H9c2 cells (1 × 10^4^/well) were seeded in μ-Slide 8-well glass bottom plates. Cells were labeled by Fluo-4 AM or JC-1 probe for 30 min after LS102 treatment according to the manufacturer’s protocols. The fluorescent intensity of images was analyzed by using the Image J software and a randomly captured method was used to make sure there were three fields per group. The fluorescence intensity of calcium, ROS were detected by the flow cytometer. All cells in the 6-well plates were collected and then suspended in a pre-warmed DMEM buffer containing CM-H2DCFDA or Fluo-4 AM calcium dye. Each group (1 × 10^4^ cells) intensity was calculated as representation.

### Quantification of Mitochondrial Length and Fragmentation

The method of mitochondrial image quantification is the same as before (Dong et al., 2016). Cells were cultured in the μ-Slide 8-well glass bottom plate. The cells were stained by 50 nM MitoView Red (GeneCopoeia, United States) after treatment at 37°C for 30 min according to the manufacturer’s recommendations. Mitochondrial morphological and structural changes were observed by a confocal microscope. The six categories of the mitochondria length are as followed: 0–1 μm, 1–2 μm, 2–3 μm, 3–4 μm, 4–5 μm, >5 μm), and the fragmentation of mitochondria confirmed as 0–1 μm (Jahani-Asl and Slack, 2007). The percentage of the 0–1 μm mitochondria was calculated by dividing the number of 0–1 μm mitochondria with the number of total mitochondria.

### Immunocytochemistry Analysis

H9c2 cells were seeded into a μ-Slide 8-well glass bottom plate at a total number of 5000 per well. After treatment, cells were first washed with PBST (0.1% Tween 20 to PBS) 3 times and then fixed with ice-cold 100% methanol for 15 min. Secondly, cells were permeabilized with 1% Triton X-100 for 20 min at room temperature. Thirdly, cells were blocked with 1% BSA/PBST buffer for 1 h and then incubated with a primary antibody of anti-Drp1 and anti-GSK-3β (1:100, Cell Signaling Technology, United States) overnight at 4 °C. Then, cells were incubated with Alexa Fluor 488-conjugated (green) and Fluor 555-conjugated (red) secondary antibody (1: 200, Cell Signaling Technology, United States) at room temperature for 2 h. DAPI (1X, Invitrogen, United States) was stained in the final step. The immunofluorescence images were captured by a confocal microscope.

### Western Blot Analysis and Co-immunoprecipitation

Cells were lysed with ice-cold RIPA buffer (Cell Signaling, MA, United States) for 20 min after washing twice with ice-cold PBS. Then the cell lysates were collected and centrifuged at 12,000 *g* for 15 min at 4°C. Equal amounts of denatured protein were separated by 8, 10, or 12% SDS-PAGE gels, and then transferred onto polyvinylidene difluoride (PVDF) membranes (Millipore Corporation, Billerica, MA, United States). After being blocked with 5% (w/v) BSA for 1 h at room temperature, the PVDF membrane was incubated with primary antibodies of anti-Drp1, anti-p-Drp1(phospho S616), anti-PI3K, anti- p-PI3K and anti-caspase3 (1:500, Cell Signaling Technology, United States); anti-p-Drp1(phospho S637) antibody (1:500, Abcam, United States); anti-GSK-3β, anti-p-GSK-3β, anti-cytochrome c, anti-Bcl-2, and anti-Bax antibodies (1:1000, Cell Signaling Technology, United States); anti-Akt and anti-p-Akt (Ser473) antibodies (1:200, Santa Cruz Biotechnology, United States); α-tubulin (1:2000, Abcam, United States) at 4°C overnight. They were then washed with PBS-T three times and subsequently hybridized with peroxidase-conjugated secondary antibodies (1:1000, Beyotime Institute of Biotechnology, Beijing, China) for 1–2 h at room temperature. Finally, the PVDF membrane was washed with PBS-T three times and was scanned by an Amersham Imager 600 (STATE of LOUISIANA, United States). β-tubulin was used to normalize the target protein expression. Proteins were denatured by heating at 100°C for 10 min in the loading buffer (contain Tris-HCl, SDS, bromophenol blue, glycerol, and β-mercaptoethanol).

An immunoprecipitation kit (Sangon Biotech, Shanghai, China) was used to detect Drp1 binding to GSK-3β, according to the manufacturers’ recommendation. The supernatant was incubated overnight at 4°C with anti-GSK-3β antibody, and then protein A agarose beads for another 2 h at room temperature. After that, the protein A agarose beads were washed six times with the 1 × IP buffer and once with the 0.1 × IP buffer. The beads were boiled in loading buffer for western blot. The PVDF membrane was incubated overnight at 4°C with the anti-Drp1 antibody.

### Statistical Analysis

All data were analyzed via GraphPad Prism 5.0 (GraphPad Software Inc, San Diego, CA, United States) and expressed as means ± SEM. Using the method of one-way analysis of variance (ANOVA) with multiple comparisons to analyze the difference between 3 or more groups. Differences were considered statistically significant at *P*-values of *P* < 0.05.

## Results

### Protective Effect of LS-102 on I/R Injury *in vivo*

**FIGURE 2:**
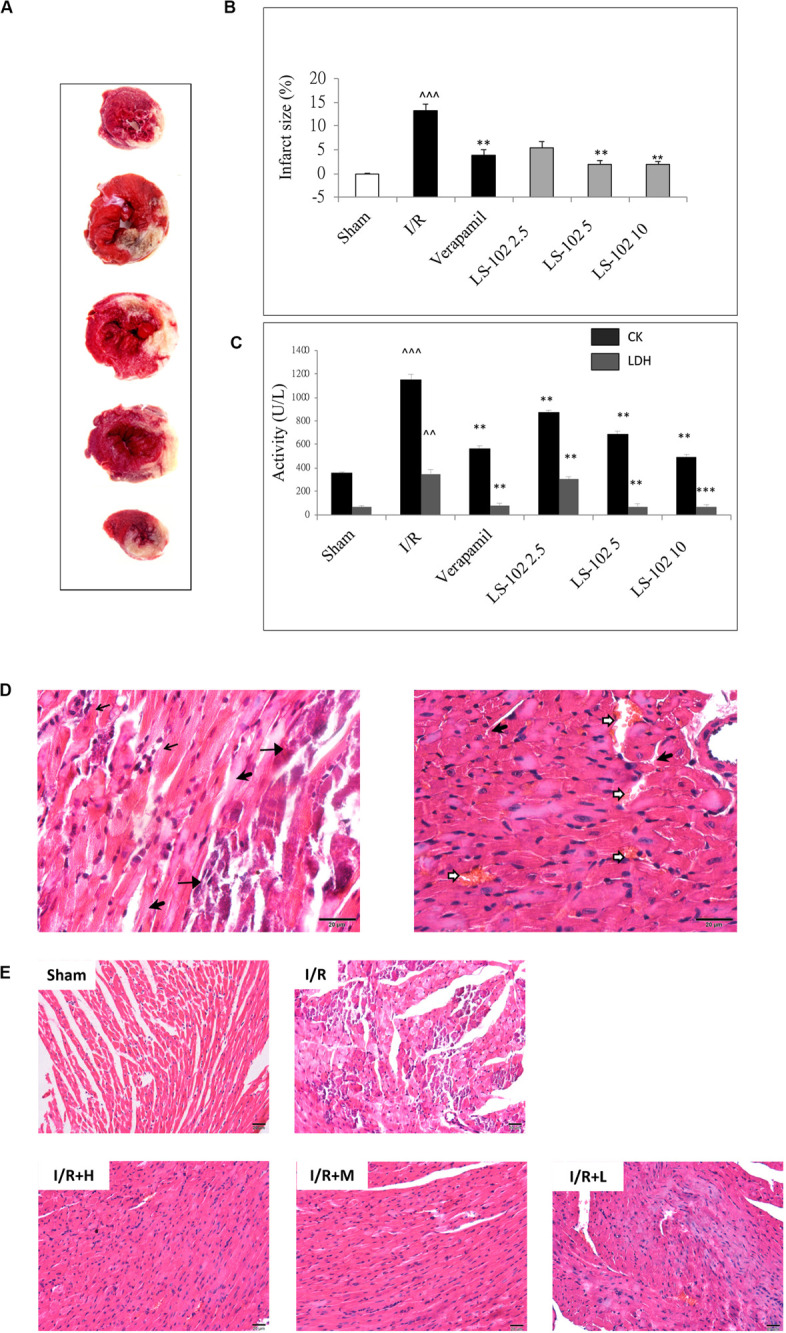
Oral-administration of LS-102 exhibited a dose-dependent relationship of cardioprotective effect in rat heart. **(A)** Representative image of TTC staining sections of the left ventricle. The viable tissue was stained as red area and the unstained pale areas were presumed to be infarcted tissue. **(B)** Bar chart of infarct size (%). **(C)** The activities of CK and LDH in serum were detected. **(D)** Representative high power views of I/R myocardial tissue slice showing neutrophil polymorphs flooding the infarcted area (thin arrow), infarcted cardiomyocytes appearing deeply eosinophilic with loss of cross striations (arrow head), prominent interstitial edema (thick arrow), RBCs in the interstitial spaces (open arrow). **(E)** Illustration of H&E staining of the myocardial tissue in different groups (200 × magnification). Sham and I/R, distilled water 8ml/kg; LS-102 2.5, LS-102 2.5 mg/kg; LS-102 5, LS-102 5 mg/kg; LS-102 10, LS-102 10 mg/kg. Data are shown as mean ± SEM, *n* = 8–10/group. ^^^*P* < 0.001, ^^*P* < 0.01, vs. Sham group, ***P < 0.001, **P < 0.01 vs. I/R group.

The measurement of myocardial infarct size is an important parameter to evaluate I/R-induced myocardial injury. Myocardial infarct size (%) was estimated by using TTC staining. Three dosages of LS-102 (2.5, 5, and 10 mg/kg) were tested in rats subjected to 30 min LAD ligation followed by 90 min reperfusion. As shown in Figures 2A,B, the myocardial infarct size in the I/R group was 13.2 ± 1.4%, with a significant increase (*P* < 0.01) when compared with the Sham group. In those that were given an oral dosage 30 min before the acute ischemia, LS-102 exhibited significant protection to rat hearts against I/R injury in a dose-dependent manner to a significant degree. In the I/R group, the serum levels of CK-MB and LDH were significantly higher than those in the sham group (*P* < 0.01). Compared with the I/R group, 5 or 10 mg/kg LS-102 treatment significantly inhibited the elevation of CK-MB or LDH activities induced by acute I/R injury (Figure 2C). A comparison between the sham and I/R group revealed histologic changes of hearts, including coagulative necrosis, neutrophil polymorphs infiltration, interstitial hypercellularity, and abundant signs of hemorrhage (Figure 2D). The cardiomyocytes in the infarct section of LV appear deeply eosinophilic with loss of the cross striation and disappearance of the nuclei. Oral administration with LS-102 at 5 or 10 mg/kg significantly reduced the necrosis of cardiomyocytes with prominent interstitial edema and inhibited the RBCs accumulation in the interstitial spaces with neutrophil polymorphs infiltration, which confirmed that LS-102 could alleviate the myocardial damage due to I/R (Figure 2E).

In our experiments, the change of ST-segment elevation, arrhythmia, and hemodynamic were investigated after I/R in rats. As shown in Table 1, the amplitude of ST-segment and heart rate were recorded and used as the indicators of changes in ischemic electrocardiograph. After coronary artery occlusion, the ST-segment markedly upregulated to 0.23 ± 0.12 mV, after ischemia compared to baseline (*P* < 0.05). Oral-treatment with LS-102 at three dosages (2.5, 5, and 10 mg/kg) markedly diminished the ST-segment elevation. Heart rate increased after ischemia in the I/R group compared to the Sham group. The heart rate decreased and was significantly inhibited after LS-102 (5 or 10 mg/kg) treatment. There was also a significantly decreased MAP in the I/R group at 90 min after reperfusion compared to the baseline. Rats treated with 5 or 10 mg/kg of LS-102 did not appear to the significant and gradual drop in MAP during the process of the I/R. Arrhythmia can be characterized by the appearance of ventricular premature beats (repeated appearance number > 3, VPB); bigeminy/trigeminy (BG/TG); ventricular tachycardia/ventricular fibrillation (appearance of bizarre, irregular; random waveform; and wandering baseline. There were no identifiable QRS complexes or P waves, VT/VF)(Garcia and Miller, 2003). The above phenomenon was investigated by II lead ECG analysis (Table 2). Compared with sham operation group, marked arrhythmia was detected (VPB, 100%; BG/TG, 75%; VT/VF, 88%) in the I/R group (*P* < 0.01; *P* < 0.01 respectively). LS-102 treatment at high dose 10 mg/kg reduced the incidence of arrhythmia ((VPB, 30% vs. 100%, *P* < 0.05; BG/TG, 0% vs. 75%, *P* < 0.01; VT/VF, 10% vs. 88%, *P* < 0.01). The above data suggested that the effect of LS-102 exerted potent protective effects against acute I/R injury in rat hearts.

**TABLE 1:** Effect of LS-102 on heart about the hemodynamic parameters.

Groups	Baseline			Ischemia			Reperfusion		
	HR (beat/min)	MAP (mmHg)	ST height (mV)	HR (beat/min)	MAP (mmHg)	ST height (mV)	HR (beat/min)	MAP (mmHg)	ST height (mV)
Sham	389 ± 25	112 ± 5	0.03 ± 0.02	395 ± 15	120 ± 12	0.05 ± 0.06	381 ± 27	112 ± 5	0.02 ± 0.04
I/R	394 ± 24	121 ± 9	0.05 ± 0.04	435 ± 23*	101 ± 14	0.23 ± 0.12*	388 ± 29	99 ± 15**	0.11 ± 0.08
I/R+L **2.5**	412 ± 19	127 ± 14	0.06 ± 0.03	446 ± 20*	100 ± 11	0.13 ± 0.09	399 ± 22	101 ± 14*	0.08 ± 0.05
I/R+M **5**	397 ± 27	127 ± 11	0.04 ± 0.03	414 ± 24	114 ± 9	0.05 ± 0.03	388 ± 36	116 ± 19	0.08 ± 0.09
I/R+H **10**	401 ± 20	111 ± 8	0.06 ± 0.04	420 ± 32	125 ± 4	0.04 ± 0.05	400 ± 33	122 ± 10	0.08 ± 0.09

The data showed the heart rate (HR), mean arterial pressure (MAP) and ST height of II lead ECG at the after 10 min of ischemia, and then 90 min of reperfusion. Values were expressed as mean ± SEM of 10 hearts from each group. Hearts underwent 30 min of ischemia and followed by 90 min of reperfusion. *P < 0.05 vs. the baseline of the same group. **P < 0.01 vs. the baseline of the same group.

**TABLE 2:** Effect of LS-102 on the incidence of cardiac arrhythmias in the rat.

Groups	Incidence (%)		
	VPB	BG/TG	VT/VF
Sham	0(0/10)	0(0/10)	0(0/10)
I/R	100(8/8)^^	75(6/8)^^	88(7/8)^^
I/R+L **2.5**	89(8/9)	56(5/9)	78(7/9)
I/R+M **5**	20(2/10)*	20(2/10)	30(3/10)
I/R+H **10**	30(3/10)*	0(0/10)**	10(1/10)**

VPB, ventricular premature beats; BG/TG, bigeminy/trigeminy; VT/VF, ventricular tachycardia/ventricular fibrillation. Numerals in parentheses represented numbers of arrhythmia hearts/number of total hearts. Values are expressed as mean ± SEM of 8–10 hearts from each group. Hearts underwent 30 min of ischemia and followed by 90 min of reperfusion. ^^*P* < 0.01, vs Sham group; *P < 0.05, **P < 0.01, vs I/R group (χ^2^ test).

### Oral-Administration of LS-102 Activated PI3K/Akt Pathways *in vivo*

**FIGURE 3:**
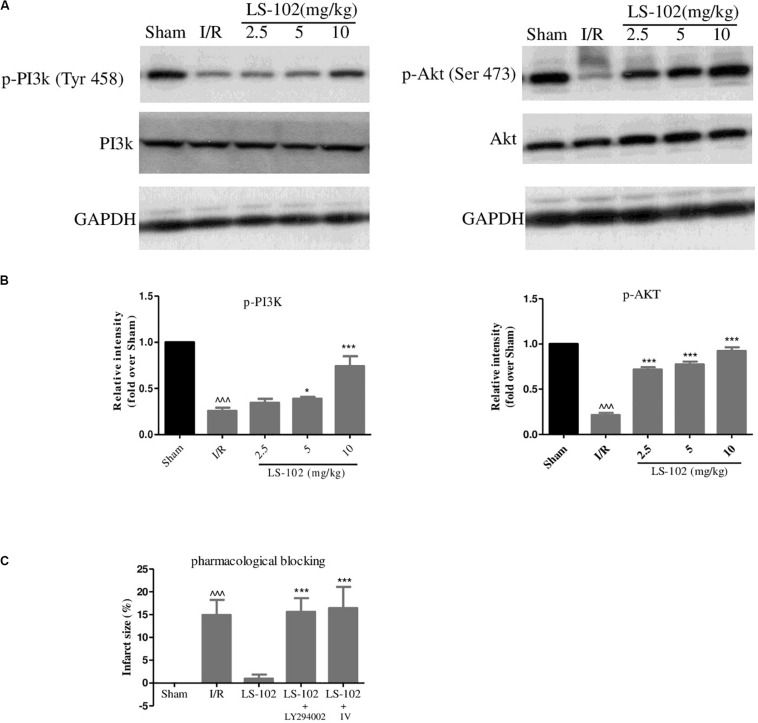
Effect of LS-102 on the PI3K/Akt signaling in rat heart. **(A)** Representative Western blot of total and phosphorylated PI3K and Akt. **(B)** Quantitative analysis of band intensity, values are expressed as mean ± S.E.M. of 4 hearts from each group. **(C)** Infarct size of a rat after the pharmacological blockage of PI3K and Akt. Sham and I/R, distilled water 8 ml/kg; LS-102, LS-102 10 mg/kg; LS-102+LY294002, LS-102 10 mg/kg+ PI3K inhibitor LY294002 0.25 mg/kg; LS-102+ IV, LS-102 10 mg/kg+Akt inhibitor IV 4mg/kg. Vehicle or LS-102 was given to rats 30 min before LAD ligation. Pharmacological inhibitors were given immediately after the administration of LS-102. Values are mean ± SEM of six rats from each group. ^^^*P* < 0.001, vs Sham group; *P < 0.05, ***P < 0.001, vs I/R group.

Previous studies have indicated that the activation of PI3K/Akt pathways has cardioprotective effects and several pharmacological agents exert the cardioprotective effects by regulating the phosphorylation of PI3K and Akt. In our study, phosphorylation of PI3K at Tyr 458 and phosphorylation of Akt at Ser 473 were investigated by Western blot analysis in the myocardium after I/R. Compared with the Sham group, the I/R group markedly down-regulated the phosphorylation of PI3K and Akt (Figures 3A,B). We have observed that LS-102 treatment (5 and 10 mg/kg) activated the expression of PI3K and Akt, which might lead to reducing cardiomyocyte apoptosis, thereby protecting myocardium from I/R-induced injury. Furthermore, we examined whether the cardioprotective effect of LS-102 is abolished by pharmacological blockage of PI3K/Akt pathways. The results (Figure 3C) demonstrated that the simultaneous administration of PI3K inhibitor LY294002 or Akt inhibitor IV abolished the infarct size reduction effect of LS-102, as evidenced by an infarct size comparable to that of the I/R group. The results of the present study showed that LS-102 significantly enhanced the expression of pro-survival PI3K/Akt, thereby conferring myocardial protection. To understand further the exact molecular mechanisms involved in the I/R-induced cardio protection, LS-102 were carried out *in vitro* in this study.

### LS-102 Attenuates Cardiomyocyte Death Induced by H/R *in vitro*

**FIGURE 4:**
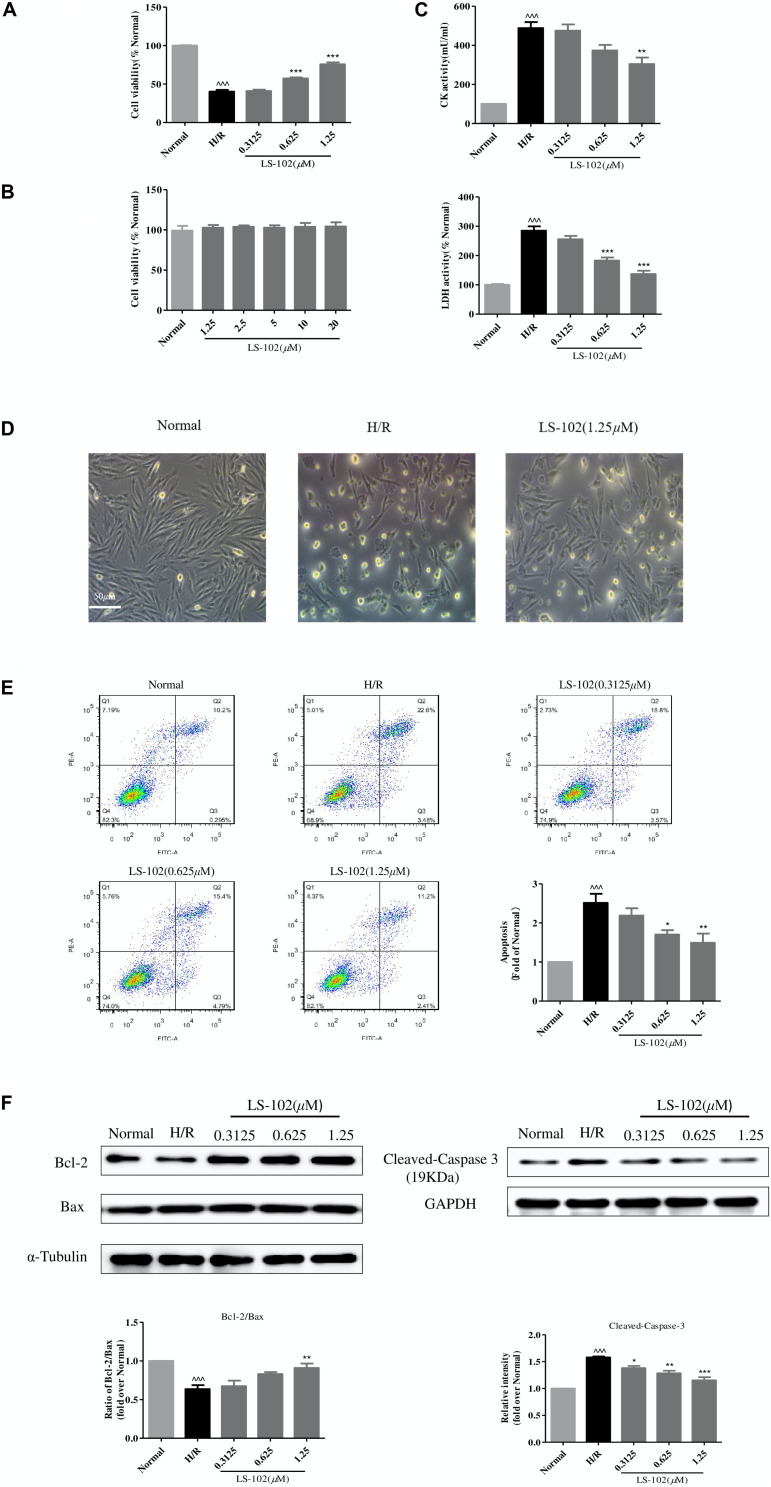
Effects of LS-102 on cell survival after H/R in H9c2 cells. **(A)** Effect of LS-102 on cell viability. **(B)** Cytotoxicity of LS-102 in H9c2 cells under normoxia condition. **(C)** Effects of LS-102 on CK and LDH activities in serum. **(D)** Representative DIC images of H9c2 cells. **(E)** Effect of LS-102 on H/R-induced H9c2 cell apoptosis. Flow cytometry was used to detect apoptotic cells which were calculated the sum of the Q2 and Q3 quadrant. **(F)** Using western blot analysis to determine the protein expression of Bcl-2, Bax, and Cleaved-Caspase-3. The ratio of Bcl-2 to Bax was calculated and normalized to the Normal group. Values are means ± SEM of three or four independent experiments. ^^^*P* < 0.001 vs. Normal group. *P < 0.05, **P < 0.01 and ***P < 0.001 vs. H/R group.

We used a well-established H/R model that caused cardiomyocyte death to investigate the protective effect of LS-102. Pretreated with LS-102 (0.3125, 0.625, and 1.25 μM) for 24 h, H9c2 cells were exposed to hypoxia (0.1% O_2_, 5% CO_2_, and 94.9% N_2_), with saturated glucose-free DMEM at 37°C for 12 h, and then 2 h reoxygenation with culture medium. H/R significantly decreased the cell viability compared with the Normal group (*P* < 0.001). LS-102 increased cardiomyocyte survival after H/R damage in a dose-dependent manner (Figure 4A). The cytotoxicity of LS-102 in H9c2 cells was also examined under normoxia. LS-102 at the concentration of 1.25–20 μM did not lead to cellular cytotoxicity or proliferation in H9c2 cells (Figure 4B). Compared with the H/R group, the inhibitory effect of LS-102 on LDH and CK activities was in a dose-dependent manner (Figure 4C). In the Normal group, cells were spindle-like and mostly arranged in parallel. Underwent H/R, cells were irregularly shrunk, rounded, and arranged in disorder. Treatment of LS-102 at 1.25 μM reduced disorderly arrangement and mostly restored the spindle-shaped morphology (Figure 4D).

Next, we examined the effects of LS-102 on apoptosis of H9c2 cells, which were analyzed by flow cytometry. As shown in Figure 4E, flow cytometric analysis demonstrated that the apoptotic cells including early apoptotic (Annexin V-FITC+/PI-) and late apoptotic cells (Annexin V-FITC+/PI+) were significantly upregulated in H/R group. LS-102 significantly reduced the percentage of apoptotic cells in a dose-dependent manner. When the treatment concentrations increased, the percentage of apoptotic cells decreased from 26.08% (H/R) to 13.61% (1.25 μM). The effects of LS-102 on caspase-3, Bax, and Bcl-2 protein expression in H9c2 cells were shown in Figure 4F. The imbalance of Bcl-2 and Bax protein expression after H/R is one of the major mechanisms underlying the cardiac apoptotic process. H/R led to significant decrease in Bcl-2/Bax expression from 1.00 (Normal) to 0.6362 ± 0.0868 (H/R) (*P* < 0.001). Cells treated with LS-102 significantly increased Bcl-2/Bax expression from 0.6362 ± 0.0868(H/R) to 0.909 ± 0.1(1.25 μM) (*P* < 0.01). Furthermore, LS-102 significantly inhibited cleaved-caspase-3 expression in a dose-dependent manner and with a maximal decrease of 1.073578 at 1.25 μM (*P* < 0.001).

### LS-102 Ameliorated Mitochondrial Dysfunction Induced by H/R *in vitro*

**FIGURE 5:**
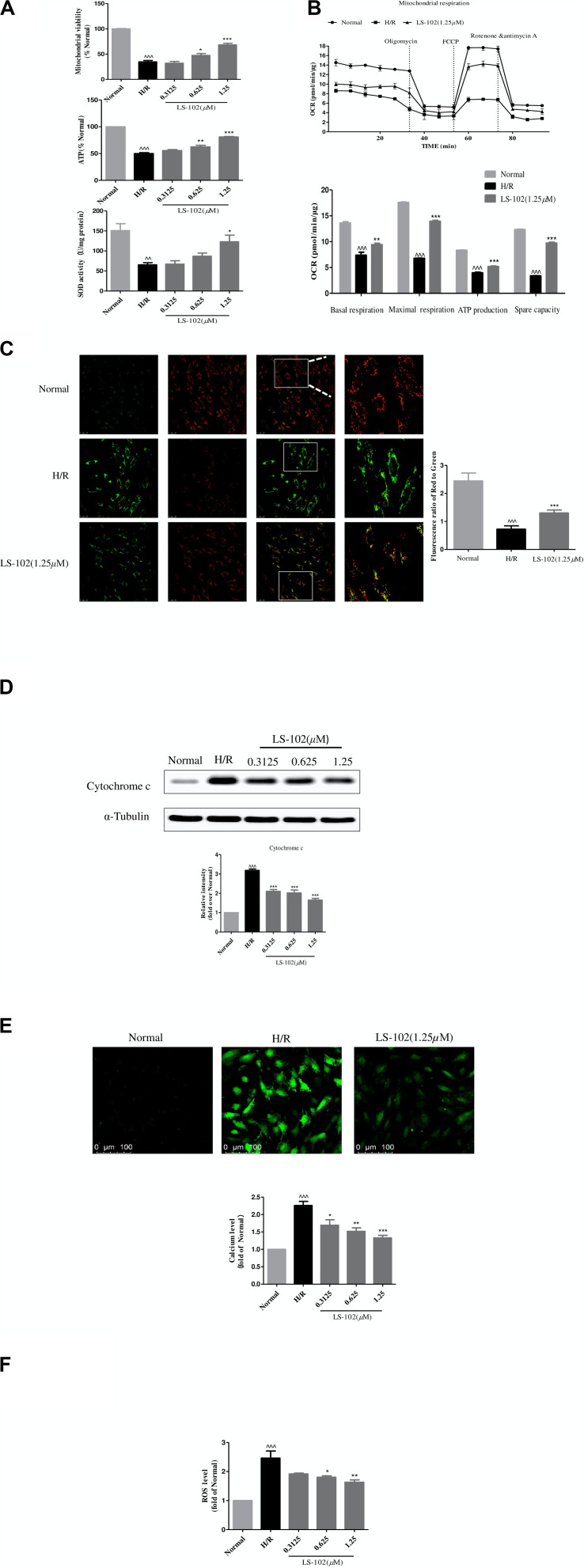
Effects of LS-102 on the mitochondrial dysfunction induced by H/R in H9c2 cells. **(A)** Effect of LS-102 on mitochondrial viability, the content of ATP, and SOD activity. **(B)** The Seahorse XFp Extracellular Flux Analyzer was used to evaluate the effect of LS-102 on oxygen consumption rate (OCR). **(C)** Representative images of JC-1 fluorescent dye stained the mitochondrial membrane potential captured by confocal microscopy with ×40 objective (Olympus). Using the fluorescence microplate assay to calculate the red/green ratio. **(D)** The protein of cytochrome c was detected by Western blot. **(E)** Representative images of Fluo-4AM stained calcium captured by confocal microscopy with ×20 objective (Olympus). The relative mean of the calcium fluorescence intensity was analyzed by flow cytometry. **(F)** The content of the ROS labeled H2DCFDA probe was analyzed by flow cytometry. The values were the means ± SEM of three or four independent experiments. ^^*P* < 0.01, ^^^*P* < 0.001 vs. Normal group. *P < 0.05, **P < 0.01, and ***P < 0.001 vs. H/R group.

The effect of pre-treatment of LS-102 on mitochondrial function after H/R is shown in Figure 5. Mitochondrial viability, ATP production, and SOD activity were reduced in H/R compared to the Normal group (*P* < 0.001, *P* < 0.001, and *P* < 0.01, respectively). Our result was indicative of the significant improvement of mitochondrial viability, ATP production, and SOD activity by LS-102 in a dose-dependent manner (Figure 5A). We also assessed mitochondrial respiration by detecting the rate of oxygen consumption. The oxygen consumption rate of H9c2 changed evidently in H/R group (Figure 5B). After LS-102 treatment, OCR (Basal), OCR (Maximal), ATP, and Spare capacity were significantly increased (*P* < 0.001 vs. H/R group, Figure 5B).

Next, we explored the changes in MMP calculated by the ratio of red to green fluorescence. The intensity of the red fluorescent signal decreased in H/R after treatment LS-102(1.25 μM) in H9c2 cells. The intensity of the red fluorescent signal increased (Figure 5C). That indicated LS-102 enhanced MMP compared to the H/R (*P* < 0.01). Subsequently, we detected the release of cytochrome c by Western blot. A significant increase of cytochrome c in H9c2 cells exposed to H/R was observed (Figure 5D). In the presence of LS-102 treatment, the release of cytochrome c significantly reduced compared to the H/R group (*P* < 0.01). Furthermore, intracellular Ca^2+^ and ROS were significantly increased more than 2 times approximately after H/R (*P* < 0.001, *P* < 0.001, vs. Normal, respectively, Figures 5E,F). LS-102 treatment significantly suppressed intracellular Ca^2+^ and ROS production in a dose-dependent manner (Figures 5E,F).

### LS-102 Regulated the Mitochondrial Dynamics by Inhibiting Drp1-Dependent Mitochondrial Fission

**FIGURE 6:**
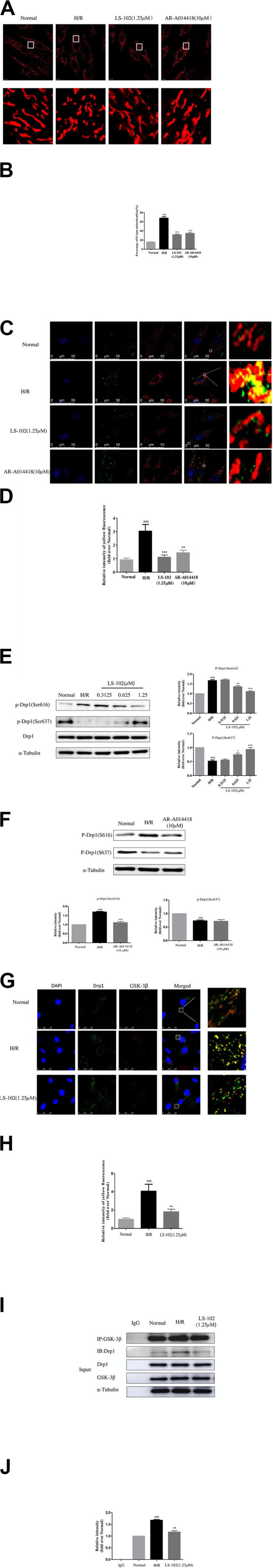
Effects of LS-102 on Drp1-dependent mitochondrial fission induced by H/R in H9c2 cells. **(A)** Representative images showing MitoTracker Red CM-H2Ros staining (MitoROS; red) were captured by confocal microscopy with ×40 objective (Olympus). **(B)** Bar graph showing the percentage of fragmented mitochondria (mitochondrial length of less than 1 μm). **(C)** Co-localization analysis of Drp1 on mitochondria using a confocal microscope with ×40 objective (Olympus). H9c2 cells were stained with MitoROS (red), anti-Drp1 antibody (green). Nucleus was trained with DAPI (blue). The yellow dots indicated Drp1(green) on the mitochondria (red) location. **(D)** Qualitative analysis of colocalization by Image J software. **(E)** Phosphorylation of Drp1 was detected by Western blot analysis and incubated with anti-phospho-Drp1(Ser637) or anti-phospho-Drp1(Ser637) antibodies, the loading control was α-Tubulin. **(F)** The protein expression of p-Drp1^Ser616^ and p-Drp1^Ser637^ after treatment of GSK-3β inhibitor AR-A014418 were determined by Western blot. **(G)** Co-localization analysis of Drp1 (green) with GSK-3β (red) were captured by confocal microscopy with × 40 objective (Olympus). **(H)** Bar chart showing yellow fluorescence intensity. **(I,J)** The protein expression of Drp1 with GSK-3β was examined by Co-immunoprecipitation (Co-IP) assay and Western blot. The relative densities of immunoreactive bands were quantified over α-Tubulin using image J software. Values are the means ± SEM of three or four independent experiments. ^^*P* < 0.05, ^^^*P* < 0.001 vs. Normal group. *P < 0.05, **P < 0.01 and ***P < 0.001 vs. H/R group.

The effect of LS-102 on mitochondrial dynamics after H/R is shown in Figure 6. H9c2 cells were stained with Mitotracker Red CM-H_2_ Ros to visualize the mitochondria. In the Normal group, mitochondria exhibited a tubular or thread-like structure, distributed uniformly throughout the cytoplasm (Figure 6A). Mitochondria were severely damaged after H/R, and the morphology changed into a small, vesicular, punctiform structure, accumulating in the perinuclear area. Both pre-treatment of LS-102 at 1.25 μM and GSK-3β inhibitor AR-A014418 at 10 μM significantly alleviated mitochondrial fragmentation and partly restored the mitochondria of rod-shape and net-like structure.

Quantitative analysis of frequency distribution and changes in the average mitochondrial length indicated that H/R significantly affected mitochondrial distribution and reduced mitochondrial length. The percentage of 0–1 μm exceeded 70%, indicating the frequency distribution of mitochondrial length among the H/R group (Figure 6B). LS-102 treatment significantly decreased the percentage of fragmented mitochondria to 36.34%. As a key regulator of mitochondrial fission, Drp1 is assembled into a spiral ring structure on the mitochondria to promote mitochondrial fission. Therefore, we conducted quantitative analysis of the diffusion and distribution of Drp1 in mitochondria. The analysis showed that H/R induced Drp1 translocation to mitochondria via immunofluorescence images. Treatment with LS-102 (1.25 μM) significantly lowered the level of Drp1 protein on the mitochondria (*P* < 0.001 vs. H/R group, Figures 6C,D). Previous studies have shown that the phosphorylation sites of Drp1 at Ser616 and Ser637 are closely related with Drp1 assemble to the mitochondrion (Jahani-Asl and Slack, 2007; Nishihara et al., 2007). As shown in Figure 6E, phosphorylation of Drp1^Ser616^ (p-Drp1^Ser616^) and dephosphorylation of Drp1^Ser637^(p-Drp1^Ser637^) were significantly up-regulated (compared to the H/R group; *P* < 0.001). In addition, LS-102 treatment significantly decreased p-Drp1^Ser616^ and increased p-Drp1^Ser637^ levels in a dose-dependent manner, when compared with the H/R group (*P* < 0.001 vs. H/R group, Figure 6E). We further investigated the underlying upstream molecular mechanisms for regulation phosphorylation of Drp1.

One kinase, GSK-3β, can regulate the mPTP or cell death under the condition of oxidative stress. The inhibition of GSK-3β effectively enhances cell vitality and anti-oxidative ability. It has to date not been reported whether, during H/R, GSK-3β contributes to the process of Drp1-dependent mitochondrial fission. In our research, the inhibition activity of GSK-3β significantly downregulated the levels of phosphorylated Drp1^Ser616^ detected (compared to the H/R group, *P* < 0.001), but the level of p-Drp1^Ser637^ expression remained unchanged, as shown in Figure 6F. GSK-3β is not only a kinase but also an anchoring protein (Harwood, 2001). We used immunofluorescence and a Co-immunoprecipitation (Co-IP) assay to confirm the interaction between Drp1 and GSK-3β protein. We found that the co-localization of Drp1 with GSK-3β upregulated in the H/R group, which was attenuated by pre-treatment of LS-102 (Figures 6G,H). Consistently, the result of Co-IP further proved that GSK-3β can bind Drp1. In the H/R group, we found Drp1 in GSK-3β-IP elutes from H9c2 cells. LS-102 treatment decreased the binding of Drp1 to GSK-3β compared to the H/R group (*P* < 0.001, Figures 6I,J). All the evidence indicates that GSK-3β can bind to phosphorylate Drp1 resulting in mitochondrial fragmentation in the model of H/R-induced H9c2 cells. LS-102 can block GSK-3β-mediated Drp1 phosphorylation and decrease the co-localization of Drp1 with GSK-3β.

### LS-102 Exerted Cardio Protection Against H/R Injury by Aactivating PI3K/Akt Pathways

**FIGURE 7:**
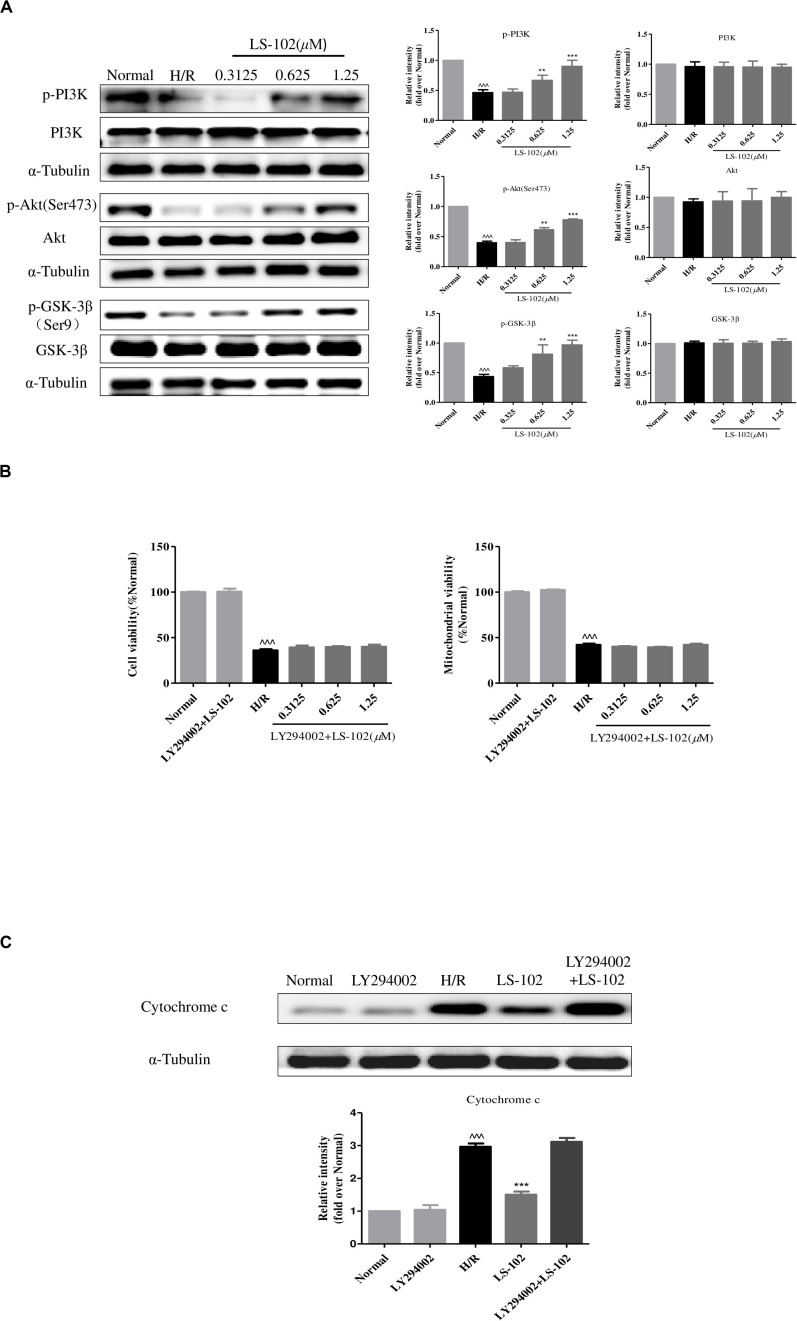
Effect of LS-102 on PI3K/Akt/GSK-3β pathway of H9c2 cells exposed to H/R. **(A)** The protein expression of PI3K, p-PI3K, Akt, p-Akt, GSK-3β, and p-GSK-3β was analyzed by Western blot. **(B,C)** PI3K inhibitor LY294002 (10 μM) altered the effect of LS-102 on cell viability, mitochondrial viability, and the cytochrome c release after H/R in H9c2 cells. Values were the means ± SEM of three independent experiments in H9c2 cells after H/R. ^^^*P* < 0.001 vs. Normal group. **P < 0.01 and ***P < 0.001 vs. H/R group.

This study has proved that GSK-3β acts as upstream signaling of Drp1 to induce mitochondrial fragmentation in H/R. Previous studies have shown that ischemic preconditioning enhances the phosphorylation and decreases the activity of GSK-3β by PI3K/Akt signal pathway. To validate the mechanisms of the underlying protective effect of LS-102, the protein expression of p-PI3K, PI3K, Akt, p-Akt, p-GSK-3β(Ser9), and GSK-3β were examined. As shown in Figure 7A, H/R-induced phosphorylation of PI3K, Akt, and GSK-3β decreased without influencing the total PI3K, Akt, and GSK-3β. Pre-treatment of LS-102 (0.3125, 0.625, and 1.25 μM) significantly increased the phosphorylation of PI3K, Akt, and GSK-3β in a dose-dependent manner (*P* < 0.001, vs. H/R). In addition, the protective effect of LS-102 was blocked by the PI3K inhibitor LY294002. LY294002, which abrogated the protective effects of LS-102 and could increase cell and mitochondrial viability, and decrease the release of cytochrome c (Figures 7B,C). All data has suggested that LS-102 increased cardiomyocyte survival in H/R by improving the mitochondria function and inhibiting Drp1-dependent mitochondrial fission via regulating the PI3K/Akt/GSK-3β pathway.

## Discussion

**FIGURE 8:**
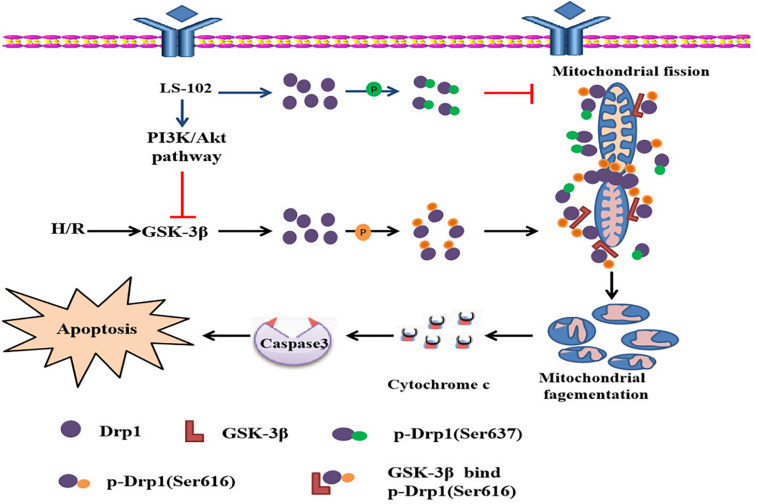
Possiblemechanism of the cardioprotective effect of LS-102 in injury of H/R.

This study is the first to prove that LS-102 exerted a powerful protective effect on I/R-induced myocardium injury, decreasing oxidative stress and apoptosis. The mechanism of cardio protection was related to the PI3K/Akt signaling pathway *in vivo* and *vitro* (Figure 8). Recently, growing evidence has indicated that maintaining the balance of mitochondrial dynamics is a potential novel therapeutic target in I/R-induced injury. We also demonstrated that LS-102 effectively prevented mitochondrial fission by decreasing Drp1 phosphorylation at Ser616 and increasing Drp1 phosphorylation at Ser637 to maintain the integrity of mitochondrial structure and function. We discovered that the Drp1 protein was involved in the downstream signaling pathway of GSK-3β, which mediated mitochondrial fragmentation by modulating Drp1^Ser616^ phosphorylation during I/R of cardiomyocytes. Therefore, PI3K/Akt/GSK-3β signaling pathway regulates cell viability and apoptosis, at least in part, depending on the GSK-3β/Drp1-mediated balance of mitochondrial dynamics.

Astragaloside IV has been widely used for treating cardiovascular diseases. Despite its efficacy and safety, AS-IV has a major problem of low bioavailability. To change this limitation, our team synthesized a new water-soluble compound LS-102. In previous research, we found that the relative bioavailability of LS-102 was twice that of AS-IV (Zhang et al., 2017; Qing et al., 2019). After conducting this research, we further elucidated the impact of LS-102 on the myocardial I/R injury to provide evidence for future clinical applications. In animal models, our results showed that LS-102 preserved LV function, attenuated the incidence of arrhythmia, reduced infarct size, and decreased the release of CK, LDH via activating the PI3K/Akt signaling pathway. Additionally, the PI3K or Akt pharmacological inhibitor abrogated the protective effects of LS-102. It is essential for the dynamic balance of mitochondrial structure and function to maintain a functional heart as a response and adaptation to metabolic or environmental stresses (Yellon and Hausenloy, 2007; Walters et al., 2012; Kloner, 2017; Forte et al., 2020; Ghosh et al., 2020; Oh et al., 2020; Tong et al., 2020). We found that LS-102 significantly enhanced the survival rate of H9c2 cardiomyocytes by modulating mitochondria in the model of H/R-induced H9c2 cell injury. LS-102 increased mitochondrial viability, OCR, and ATP content. Meanwhile, LS-102 reduced free radical injury by decreasing ROS production, calcium overload, and increasing the activity of SOD. We also detected the influence of LS-102 on mitochondrial-related apoptosis pathways. LS-102 prevented the mitochondrial apoptosis pathway by stabilizing MMP, inhibiting the release of cytochrome c, decreasing the ratio of Bax/Bcl-2, and inhibiting the cleavage of Caspase-3. The protective effects of LS-102 are mainly attributed to the activation of the PI3K/Akt signaling pathway. Moreover, the PI3K inhibitor (LY294002) abolished the effect of LS-102 on H/R-induced cardiomyocytes, mitochondrial viability, and the release of cytochrome c, which was further confirmed it.

Mitochondrial fission can be described as mitochondrial dynamics, that play essential roles in maintaining mitochondrial homeostasis. Drp1-dependent mitochondrial excessive fission cause mitochondrial fragmentation, ROS production, depolarization of the MMP, the release of cytochrome c, and the susceptibility of cell apoptosis, which is a key player in the pathogenesis of I/R (James et al., 2003; Karbowski et al., 2004; Lee et al., 2004; Hamacher-Brady et al., 2006; Ong et al., 2010; Sharp et al., 2014). Reports from *in vitro* and *in vivo* studies have shown that pharmacologic or genetic inactivation of Drp1 intervention can prevent mitochondrial fission in myocardial I/R injury models (Ong et al., 2010; Disatnik et al., 2013; Sharp et al., 2014; Maneechote et al., 2018). As far as we know, there is currently limited research about traditional Chinese medicine and the ability of these alternative drugs to regulate mitochondrial dynamics. In the present study, we observed that mitochondrial morphologies were changed into fragment or punctiform and the length of mitochondria was short compared with the normal mitochondria after H/R. These results were in agreement with our previous experimental and theoretical results (Dong et al., 2016). While LS-102 increased the length of mitochondrial and preserved mitochondrial morphologies and structure, LS-102 resisted and H/R-triggered the mitochondrial dynamic abnormality by preventing mitochondrial fragmentation. Although inhibition of the activity of Drp1, by Mdivi-1, or Drp1 siRNA, or TH or FK506 can preserve mitochondrial networking and ultrastructure to protect the heart, it can also have severe side effects or toxicity. LS-102 as natural products not only have less toxicity or side effects but they also keep a balance in the mitochondrial dynamic by decreasing the activity of Drp1. LS-102 provides us with a great source for screening new compounds in inhibition of the Drp1 activity.

Drp1-dependent mitochondrial fission is the primary reason to cause excessive mitochondrial fragmentation, which is regulated by Drp1. The activity of Drp1 GTPase enzyme and the ability of Drp1 mitochondrial translocation are mainly associated with the phosphorylation sites of Drp1 (Otera et al., 2013). It has been proven that Drp1 has many phosphorylation sites including serine 585 (Ser585), 616 (Ser616), 637 (Ser637), 656 (Ser656), and 693 (Ser693) (Cribbs and Strack, 2007; Cereghetti et al., 2008; Chou et al., 2012; Li and Li, 2015). Interestingly, Drp1 phosphorylation has opposing functional and morphological effects on different serine residues. Upregulated Drp1 activity and stimulated Drp1 mitochondrial translocation could be alerted via inhibition of PKCε and Ser637 dephosphorylation by activation of calcineurin or activation of Cdk1 and PKCδ during I/R (Cribbs and Strack, 2007).

In our study, we observed that Drp1 phosphorylation both at Ser616 and dephosphorylation at Ser637 elevated in H9c2 cells of H/R. Phosphorylation at Drp1 pSer616 site and dephosphorylation of Drp1 at Ser 637 site made Drp1 translocate to mitochondria and induced mitochondrial degeneration/fragmentation, which could be reversed by LS-102, as it prevented Drp1-dependent mitochondrial fission and balanced mitochondrial dynamics to maintain the integrity of mitochondrial structure and function. Meanwhile, we also proved that LS-102 exerted cardio protection via activation of the PI3K/Akt/GSK-3β signaling pathway. In H/R-induced mitochondrial dysfunction, these results indicate that GSK-3β could be an upstream signaling mechanism for increasing the activity of Drp1.

Current evidence suggests that GSK-3β is involved in mitochondrial dysfunction-mediated cell apoptosis. Inhibition of GSK-3β activity has been implicated as a cardioprotective mechanism in I/R injury (Linseman et al., 2004; Watcharasit et al., 2008). Initially, Hong et al. (1998) demonstrated that GSK-3β interacted with the C-terminal GED domain of Drp1 in yeast and mammals (Hong et al., 1998; Chen et al., 2000). In a separate study of 2013, Chou et al. confirmed that GSK3β-mediated phosphorylation at the Ser693 site induced prolongation of mitochondrial morphology and presented an anti-apoptotic effect on H_2_O_2_-induced apoptosis (Chou et al., 2012). In addition to regulating the phosphorylation of Drp1 translocation to mitochondria, GSK-3β can also up-regulate the expression of Drp1. Wu et al. (2013) also found that GSK-3β facilitated cell death via the upregulation of Drp1 expression and mitochondrial fission (Wu et al., 2013). There is evidence that I/R induced GSK-3β activation (Zhai et al., 2011). However, little is known about the relationship between GSK-3β and mitochondrial fragmentation in H9c2 cells during H/R injury. In this study, we found that the GSK-3β inhibitor (AR-A014418) prevented H/R-induced Drp1 translocation to mitochondria and mitochondrial fragmentation. Furthermore, the inhibition of GSK-3β activity successfully prevented Drp1 phosphorylation at Ser616 in H9c2 cells. These results are the first to discover that GSK-3β-induced phosphorylation at Ser616 may be associated with mitochondrial fragmentation and correlated with weakened resistance to injury.

In 2017, Zhou et al. (2017) showed that increasing the Drp1 binding to GSK-3β might be an important factor in mPTP opening after cerebral I/R injury. In our study, we extend these findings and proved that GSK-3β bound Drp1 increased mitochondrial fragmentation, using Co-IP method. GSK-3β is one of the targets underlying I/R induced mitochondrial abnormalities and medication for GSK-3β-induced Drp1 phosphorylation may be the new target for the treatment of I/R injury. Interestingly, LS-102 not only blocked GSK-3β-mediated Drp1 phosphorylation but also decreased co-localization of Drp1 with GSK-3β. LS-102 prevented Drp1-mediated mitochondrial fission, partly because it blocked GSK-3β-induced Drp1 phosphorylation and exerted protective effects against I/R injury.

## Conclusion

This study had outlined evidence that LS-102 is affected by ischemia-reperfusion both *in vivo* and *in vitro*, providing acute protection to the heart. Firstly, LS102 effectively protected the degree and scope of I/R induced injury in rats by reducing infarct size, the incidence of arrhythmia, the activity of CK, LDH, and preserving the LV function. Importantly, mitochondrial dysfunction is a key feature of ischemia reperfusion. Our study discovered that the LS-102 treatment ameliorated oxidative stress, apoptosis, and imbalance of mitochondrial dynamic, at least in part, associated with the PI3K/Akt signaling pathway in vivo and vitro. Moreover, this protection is related to the fact that it inhibits mitochondrial fission and maintains the integrity of mitochondrial structure and function. It requires inhibiting the phosphorylation of Drp1 via the activation of the GSK-3β pathway, thus it may provide a new pharmacological approach for the future development of therapeutic strategies.

## Ethics Statement

The animal study was reviewed and approved by State Key Laboratories for Quality Research in Chinese Medicines, Macau University of Science and Technology.

## Author Contributions

PL and L-SQ designed the study and obtained the financial support. LC, X-YC, and Q-LW performed the experiments. LC wrote the manuscript. HZ and S-JY carried out the data analysis. LC, L-SD, and X-YC critically reviewed the manuscript. All authors read, discussed, and accepted the manuscript.

## Conflict of Interest

The authors declare that the research was conducted in the absence of any commercial or financial relationships that could be construed as a potential conflict of interest.
